# The CCAAT/Enhancer-Binding Protein Family: Its Roles in MDSC Expansion and Function

**DOI:** 10.3389/fimmu.2019.01804

**Published:** 2019-07-31

**Authors:** Wenxin Wang, Xueli Xia, Lingxiang Mao, Shengjun Wang

**Affiliations:** ^1^Department of Laboratory Medicine, The Affiliated People's Hospital, Jiangsu University, Zhenjiang, China; ^2^Jiangsu Key Laboratory of Laboratory Medicine, Department of Immunology, School of Medicine, Jiangsu University, Zhenjiang, China

**Keywords:** myeloid-derived suppressor cells, C/EBPs, transcription factors, immunosuppression, differentiation

## Abstract

Immunosuppressive cells have been highlighted in research due to their roles in tumor progression and treatment failure. Myeloid-derived suppressor cells (MDSCs) are among the major immunosuppressive cell populations in the tumor microenvironment, and transcription factors (TFs) are likely involved in MDSC expansion and activation. As key regulatory TFs, members of the CCAAT/enhancer-binding protein (C/EBP) family possibly modulate many biological processes, including cell growth, differentiation, metabolism, and death. Current evidence suggests that C/EBPs maintain critical regulation of MDSCs and are involved in the differentiation and function of MDSCs within the tumor microenvironment. To better understand the MDSC-associated transcriptional network and identify new therapy targets, we herein review recent findings about the C/EBP family regarding their participation in the expansion and function of MDSCs.

## Introduction

In the tumor microenvironment, immunosuppressive cells function to inhibit tumor immune responses and promote tumor immune evasion. These cells include regulatory T cells (Tregs), regulatory dendritic cells (regDCs), tumor-associated macrophages (TAMs), and myeloid-derived suppressor cells (MDSCs). MDSCs are a heterogeneous population of immunosuppressive cells that comprise myeloid progenitor cells and immature myeloid cells and exert immunosuppressive and pro-tumorigenic effects (1, 2). Under physiological conditions, immature myeloid cells (IMCs) are generated in the bone marrow and eventually differentiate into mature dendritic cells, macrophages, or granulocytes (1). In contrast, under pathological conditions, IMC differentiation into mature myeloid cells is blocked, resulting in the accumulation of MDSCs and the upregulation of immune suppressive factors, such as arginase, nitrogen species, and reactive oxygen species (3–5). MDSCs can be divided into 2 major populations: polymorphonuclear MDSCs (PMN-MDSCs), which have a CD11b^+^Ly6G^+^Ly6C^lo^ phenotype in mice and a CD11b^+^ CD14^−^CD15^+^ (or CD66b^+^) phenotype in humans; and mononuclear MDSCs (M-MDSCs), which are defined as CD11b^+^Ly6G^−^Ly6C^hi^ in mice and CD11b^+^CD14^+^HLA-DR^−/lo^ CD15^−^ in humans (4, 6, 7). In addition, Lin ^−^ (including CD3, CD14, CD15, CD19, CD56) HLA-DR^−^CD33^+^ cells have been properly defined as “early-stage MDSC” (eMDSC) in humans, and the mice equivalent is not clearly determined. In particular, functional characteristics and biochemical and molecular characteristics are also necessary to identify cells as MDSCs (7).

It is known that both the expansion and activation of MDSCs are under precise regulation, and multiple factors have been documented after active investigation in recent years, especially transcription factors (TFs). To modulate MDSCs, TFs bind to DNA to induce the transcription of multiple target genes, taking part in the mediation of MDSCs. Such TFs include CCAAT/enhancer-binding proteins (C/EBPs), signal transducers and activators of transcription (STATs), interferon regulatory factor (IRF), hypoxia-inducible factors(HIF), and nuclear factor kappa B (NF-κB) (8–12). In recent years, many reports have focused on the C/EBP family, particularly C/EBPβ, which may play a fundamental role among all TFs. Here, we review the critical role of the C/EBP family in the regulation of MDSCs and explore new therapeutic targets in the tumor microenvironment.

## The C/EBP Family: The Basics

The C/EBP family consists of six structurally and functionally homologous transcription factors: C/EBPα, C/EBPβ, C/EBPγ, C/EBPδ, C/EBPε, and CHOP. C/EBPs are modular proteins composed of a transactivation domain (TAD), a regulatory domain, a basic DNA-binding domain, and a “leucine zipper” domain, by which family members can form homodimers and heterodimers (13, 14). Following dimerization, the DNA-binding domain recognizes and binds to the palindromic sequence ^A^/_G_TTGCG^C^/_T_AA^C^/_T_ to regulate expression of target genes (15).

The unique structure and precise regulation of C/EBPs determine their complex and significant functions in multiple cells (16). A variety of extracellular signals can act as activators or inhibitors of C/EBPs via distinct signal transduction pathways, and expression and activation of C/EBPs are regulated in a complex manner by posttranslational modifications and protein–protein interactions (16–18). Following activation, several classes of genes are induced or repressed by C/EBPs, including cytokines and chemokines, their respective receptors, proinflammatory factors, differentiation-related markers and metabolic enzyme genes, with diverse effects on distinct cells.

## The Role of the C/EBP Family in Myelopoiesis

The C/EBP family plays a central role in diverse pathophysiological events, including liver metabolism, adipogenesis, hematopoiesis, inflammatory processes, and tumorigenesis (19–22). Specifically, the C/EBP family is involved in regulating the development of myelomonocytic cells as well as the specific functions of this cell type (19, 23, 24). The role of C/EBPα in the development of leukemia has been extensively investigated, and C/EBPβ has also been directly connected to the development of different myelomonocytic leukemias. In addition, C/EBPβ^ko^ mice are characterized by hyperplastic hematopoiesis and hypermyeloproliferation. Interestingly, the complete absence of C/EBPβ differentially affects the proliferation of cells at distinct developmental stages or further differentiated cells.

Among the C/EBP family, C/EBPα, and C/EBPβ have fundamental roles in myelopoiesis. Although C/EBPα and C/EBPβ share many common target molecules, they exhibit different abilities, especially under emergency conditions. First, C/EBPα strongly inhibits the cell cycle, represses proliferation, and induces granulocyte differentiation. In contrast, C/EBPβ has less of an inhibitory effect and is associated with proliferation and differentiation. Second, C/EBPα and C/EBPβ are required for steady-state and emergency granulopoiesis, respectively. One study reported that all members of the C/EBP family, except for C/EBPβ, were downregulated in response to cytokine stimulation under emergency conditions (25). Third, C/EBPα functions as the “granulopoiesis molecular switch” at the early stage of myeloid differentiation, whereas the level of C/EBPβ expression increases dramatically at later stages of differentiation (26). Nonetheless, a recent study indicated a role for C/EBPβ in regulating hematopoietic stem cells under emergency conditions (27). Considering their homology, the different roles of C/EBPα and C/EBPβ in myelopoiesis may also be true in humans.

As MDSCs can be considered to be an abnormal myeloid population under pathological conditions, we explore whether C/EBPs also contribute to MDSC processes.

## The C/EBP Family Functions in MDSCs

### C/EBPα

C/EBPα was first described in 1986 as the founding member of the C/EBP family. Two isoforms of C/EBPα are generated from its mRNA by a ribosomal scanning mechanism. The full-length protein is 42 kDa, containing a negative regulatory subdomain, and the other isoform is a shorter 30-kDa protein with altered transactivation potential (13).

C/EBPα promotes differentiation and is necessary for normal granulocyte expansion (20–22). C/EBPα, a differentiation factor, and c-myc, a proliferative factor, act in opposition to each other. C/EBPα and its negative regulation of c-myc allow for early myeloid precursors to enter a granulocytic differentiation pathway, thus indicating their potential role in the differentiation of MDSCs. In addition, an E2F binding site in the c-myc promoter acts as a *cis*-acting element critical for C/EBPα negative regulation. C/EBPα and pRb function in parallel to repress E2F, providing a potential mechanism for C/EBPα-mediated growth inhibition (28, 29). In accordance with this finding, C/EBPα knockout mice show a reduction in mature neutrophil/monocyte cells due to blockade of the transition between CMPs (common myeloid progenitors) and GMPs (granulocyte macrophage progenitors) (30). Similarly, PU.1 acts with C/EBPα to complete the transition from CMPs to GMPs and regulate expression of myeloid-specific genes, such as myeloperoxidase as well as colony-stimulating factors (CSFs), cytokines, and their receptors (31).

As shown in a study by Hegde et al. (32), miRNA-690 is highly overexpressed in Δ(9)-tetrahydrocannabinol-induced MDSCs (THC-MDSCs). C/EBPα was identified as a potential functional target of miR-690, and downregulation of C/EBPα likely plays a crucial role in myeloid expansion and differentiation and in cannabinoid-induced immunosuppression (32).

### C/EBPβ

C/EBPβ (also known as NF-IL6, IL6-DBP, CRP2, NF-M, AGP/EBP, ApC/EBP, or TCF5) has three isoforms: liver-enriched activator proteins LAP^*^, LAP, and liver-enriched inhibitory protein LIP (6). A difference of 21 amino acids (23 in human proteins) in the TAD region between LAP^*^ and LAP leads to distinct physiological roles. LAP^*^ participates in terminal differentiation, whereas LAP and LIP promote cell proliferation and tumor progression. The truncated LIP, which lacks the entire transactivation domain, attenuates transcriptional activity via heterodimerization with LAP(LAP^*^). Proper ratios of all three isoforms are critical for normal cell growth and development (13, 14, 19, 33).

**Figure 1:**
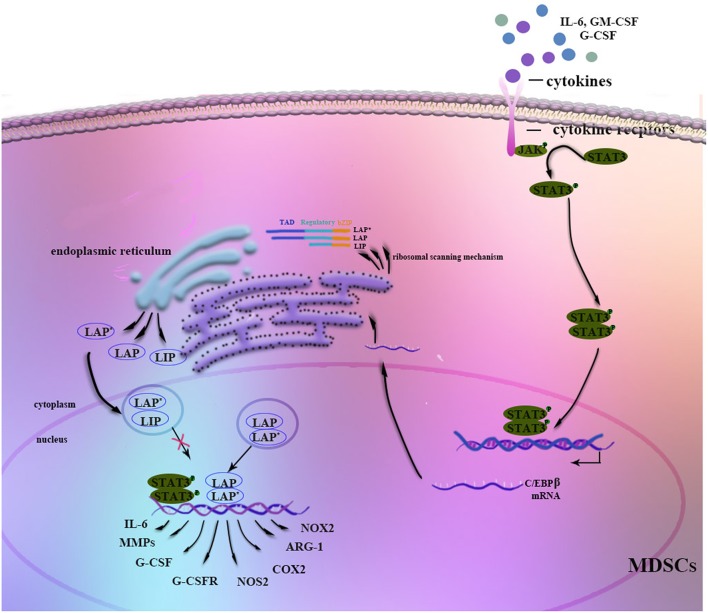
Regulation of C/EBPβ in MDSCs. Stimulation by G-CSF, GM-CSF, IL-6, or other factors strongly induces C/EBPβ expression in MDSCs. The JAK-STAT3 signaling pathway may function as an upstream regulator of C/EBPβ. Three isoforms of C/EBPβ are generated from its mRNA through a ribosomal scanning mechanism. In addition, C/EBPβ cooperates with p-STAT3 or NF-κB by sharing the same target genes. On the one hand, C/EBPβ LAP* and LAP function as transcriptional activators of the expression of immunosuppressive genes such as Arg-1, NOS2, NOX2, or COX2, whereas LIP attenuates function by blocking LAP and LAP* signaling. On the other hand, C/EBPβ affects the differentiation and expansion of MDSCs by regulating IL-6, CSFs, CSFRs, MMPs, or other factors.

Stimulation by IL-6, IFN-γ, TNF, and IL-1 induces C/EBPβ mRNA and protein expression as well as C/EBPβ DNA-binding activity, suggesting a role in mediating the inflammatory response (19, 34). Moreover, C/EBPβ LAP^*^ and LAP function as transcriptional activators of inflammation-linked genes, such as IL-6, TNF, and G-CSF, while LIP reduces inflammation by blocking LAP and LAP^*^ signaling (13). Considering its role in the inflammatory response, C/EBPβ induction by cytokines, especially IL-6, is crucial for the regulation of MDSC differentiation and function in the tumor microenvironment (35–37) (Figure 1). Studies have shown that C/EBPβ activates miR-21 and miR-181b expression, inducing NFI-A to support MDSC expansion in the bone marrow and spleens of septic mice (38, 39). In contrast, C/EBPβ deficiency affects the differentiation of MDSCs, mostly M-MDSCs (35). IL-6-mediated lnc-C/EBPβ binds to LIP to inhibit activation of LAP and to downregulate expression of immunosuppressive genes such as arginase-1 (Arg-1), NO synthase 2 (NOS2), NADPH oxidase 2 (NOX2), and cyclooxygenase-2 (COX2) (37). Another study by Gao et al. demonstrated the relationship among lnc-chop, C/EBPβ and CHOP, with lnc-C/EBPβ and lnc-chop cooperatively regulating the balance among the three C/EBPβ isoforms. Furthermore, C/EBPβ might also modulate MDSCs by controlling myeloid cell survival (36, 40).

It is known that C/EBPβ is indispensable for myelopoiesis and MDSC processes. However, it is not clear at which stage C/EBPβ specifically affects myelopoiesis. C/EBPα enables the granulocyte-lineage specific transition from CMPs to GMPs; beyond the GMP stage, C/EBPα is dispensable, with C/EBPβ playing the predominant role (41). One previous study indicated that RORC1 (retinoic-acid-related orphan receptor 1) contributes to the accumulation of PMN-MDSCs (39, 40, 42, 43). Additionally, RORC1 orchestrates myelopoiesis by promoting C/EBPβ and suppressing SOCS3 at myelocyte (MC) and metamyelocyte (MM) stages. Moreover, a study by McClure et al showed that in late sepsis, binding of pSTAT3 and C/EBPβ to the miR-21 and miR-181b promoters is restricted to the CD31^+^ subset of Gr1^+^CD11b^+^ MDSCs, a more immature stage of Gr1^+^CD11b^+^ cells (8).

The different roles of C/EBPβ isoforms require further research. The decrease in the LAP/LIP ratio has been strongly associated with proliferation in hematopoietic cells (44). LAP^*^ and LAP with transcriptional activation domains are able to promote differentiation of the granulocytic lineage in murine bone marrow cells (45), though the study by Sonda et al. reported the opposite results (46). The role of miR-142-3p in the regulation of myeloid differentiation has recently been investigated, and Sonda et al. detected downregulation of LAP* in miR-142-3p-overexpressing BM-MDSCs. Intriguingly, BM cells induced toward granulocytic differentiation show a strong decrease in LAP^*^ content, whereas macrophage differentiation causes LAP^*^upregulation.

Thus, it is clear that there are two different subsets of MDSCs. The independent roles of C/EBPβ in MDSC subsets and in the selective differentiation of MDSCs need to be explored. As shown by Sonda et al., the question of whether the LAP^*^/LIP ratio is able to determine the differentiation fate of M-MDSCs or PMN-MDSCs remains unanswered (46). RORC1 might also orchestrate the M-MDSC transition to TAMs by regulating C/EBPβ (39, 40, 42, 43).

### C/EBPδ

C/EBPδ is encoded by the single-exon CEBPD gene, and only one full-length protein is known (47). Although C/EBPδ has diminished DNA-binding activity compared to C/EBPα and C/EBPβ, it is also associated with diverse gene expression and functions (13).

C/EBPδ modulates many biological processes in a cell-type and context-dependent manner (47). Because of its roles in DNA repair, genomic stability, cell growth arrest, cell death, cell differentiation and the innate immune response, C/EBPδ might function as a tumor suppressor in the early stages of tumor development (47). In contrast, C/EBP-δ is thought to be an important factor in regulating gene transcription of various inflammatory cytokines and acute-phase proteins, such as COX-2, iNOS, G-CSF, IL-1β, IL-6, and TNF-α (48). C/EBPδ also causes upregulation and persistence of HIF-1α under hypoxic conditions (49). Because inflammation and hypoxia are two conditions closely associated with tumor development, C/EBP-δ might also have a positive role in tumorigenesis. Additionally, recent studies have reported its functions in lymphangiogenesis, angiogenesis, and tumor metastasis (50, 51).

C/EBPδ is expressed by differentiated granulocytes/neutrophils, with a specific role in emergency myelopoiesis under tumor conditions (23, 24, 50, 51), and a recent study suggested that C/EBP-δ positively regulates MDSC expansion and endothelial VEGFR2 expression in tumor development (50). The pathological upregulation of C/EBP-δ in MDSCs constitutes another mechanism for tumor growth and progression.

### CHOP

C/EBP-homologous protein (CHOP; encoded by Ddit3 and also known as CHOP−10 and Gadd153) typically leads to apoptosis and correlates with tumor progression (52). Increased expression of CHOP is observed at tumor sites and in malignant cells and infiltrating myeloid cells. Recent studies have shown that CHOP might have a critical role in the expansion and function of tumor-infiltrating MDSCs and may also be partly responsible for the short lifespan of MDSCs (53, 54).

CHOP is thought to lack DNA-binding activity and cannot form homodimers, but it can form heterodimers with other C/EBP family proteins to affect their activities (55). Thevenot et al found that chop upregulation in MDSCs was induced by tumor-induced ROS or PNT. CHOP could interact with C/EBPβ isoform LIP to regulate C/EBPβ isoform LAP activity, promoting MDSC immunosuppressive functions. In contrast, MDSCs derived from Chop-deficient mice acquired a DC-like phenotype and were able to stimulate immune response, reversing MDSC activity (53). A study by Gao et al. showed similar results. IL-6–mediated lnc-chop bound with both CHOP and LIP to promote activation of LAP. Meanwhile, lnc-chop also promoted the accumulation of H3K4me3 in the promoter region of immunosuppressive genes. Interestingly, lnc-chop might also affect MDSCs to differentiate into M-MDSCs (monocytes/macrophages) (56).

CHOP is thought to lack DNA-binding activity and cannot form homodimers, but it can form heterodimers with other C/EBP family proteins to affect their activities (55). Thevenot et al found that CHOP upregulation in MDSCs is induced by tumor-induced reactive oxygen species (ROS) or PNT. CHOP is able to interact with the C/EBPβ isoform LIP to regulate the activity of the C/EBPβ isoform LAP, promoting MDSC-immunosuppressive functions. In contrast, MDSCs derived from CHOP-deficient mice acquire a DC-like phenotype and are able to stimulate the immune response, reversing MDSC activity (53). Additionally, a study by Gao et al. reported similar results. IL-6-mediated lnc-chop binds to both CHOP and LIP to promote activation of LAP, and lnc-chop promotes accumulation of H3K4me3 in the promoter region of immunosuppressive genes. Interestingly, lnc-chop might also prompt MDSCs to differentiate into M-MDSCs (monocytes/macrophages) (56).

Overall, C/EBPε is known to be a master regulator of terminal differentiation in granulocytes (36, 40). However, the relationship between C/EBPε and MDSCs remains to be clarified.

## Co-operation Among C/EBPs

**Figure 2:**
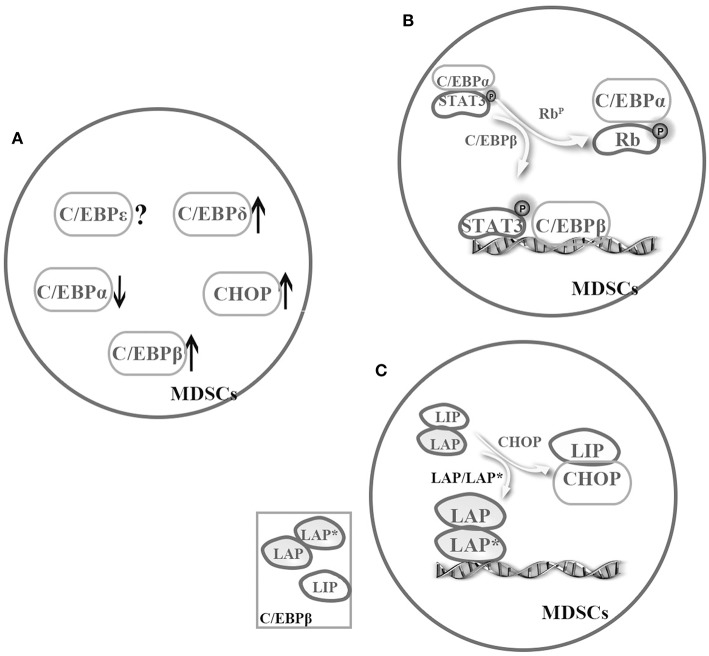
Cooperation among the C/EBP family members in MDSCs. The C/EBP family members cooperatively regulate MDSCs. **(A)** Under pathological conditions, C/EBPα might relatively downregulate, but C/EBPβ, C/EBP-δ, and CHOP might strongly upregulate in MDSCs (4, 25, 32, 35–37, 50, 57). **(B)** There is a possible switch between C/EBPα and C/EBPβ in MDSCs, that is sufficient to turn on or off target promoter (4, 8). **(C)** C/EBPβ exists as three isoforms, and one cooperation model is promoting activation of LAP or LAP*. For example, CHOP binds to LIP and to sequester LAP or LAP* in MDSCs (53, 56).

Regulation by TFs becomes more complex when considering cooperation among these regulators. According to the previous discussion, we know that C/EBPα might relatively downregulate, but C/EBPβ, C/EBP-δ, and CHOP might strongly upregulate in MDSCs under pathological conditions (4, 25, 32, 35–37, 50, 57). Although almost C/EBP family members are involved in the regulation of MDSCs, C/EBPβ is even more important. Especially, C/EBPβ exists as three isoforms, and one co-operation model is promoting activation of LAP or LAP^*^. As mentioned earlier, CHOP binds to LIP and to sequester LAP or LAP^*^ in MDSCs (53, 56) (Figure 2). In general, more studies are required to unveil cooperation among C/EBPs family.

Of the various TFs, C/EBPβ and STAT3 are mainly implicated in MDSC expansion and function. Stimulation by G-CSF, GM-CSF, IL-6, IL-1, or factors strongly induce C/EBPβ expression in MDSCs (35–38), and the JAK-STAT3 signaling pathway can function as an upstream regulator of C/EBPα, C/EBPβ, NF-κB, and IRF-8, among others (8, 12, 57). A study by Zhang et al. showed that STAT3 activates its downstream target C/EBPβ with relative downregulation of C/EBPα in response to G-CSF, ultimately regulating c-myc activity, which partly accounts for CD11b^+^Gr1^+^ cell expansion (4).

Nonetheless, the question of how C/EBPs function with other TFs in a cooperative manner remains unanswered. C/EBP family members have similar abilities to regulate transactivation in MDSCs by forming homologous or heterogeneous dimers. In addition, C/EBPs not only act as downstream targets of STAT3 but also cooperate with STAT3 and NF-κB by sharing target genes involved in proliferation, survival, chemokine and cytokines. In addition, several mutagenesis studies have shown that C/EBPs and NF-κB binding sites are necessary for transactivation of the IL-6 gene. Similarly, another study suggested that pRb regulates the miR-21 and miR-181b promoters during sepsis by binding and sequestering the C/EBPα protein, which allows C/EBPβ and pSTAT3 to bind to these miR promoters (8). These findings suggest a possible switch between C/EBPβ and C/EBPα that is sufficient to turn on or off target promoter (4, 8) (Figure 2). In addition, C/EBPs can interact with other TFs or proteins such as Rb, E2F, PU.1, runt-related transcription factor (Runx) proteins, p300/CREB-binding protein (CBP), and death-associated protein 6 (Daxx) in distinct cells (19), though whether the interaction exists in MDSCs remains to be further investigated.

## Conclusions

It is evident that C/EBP family members control the expansion and functional features of MDSCs. The relationship between C/EBPs and MDSCs can be demonstrated in both mice and humans. However, most reports have concentrated on C/EBPβ. Thus, more exploration of other C/EBP family members and their upstream or downstream molecules is needed. In addition, we should pay attention to the different roles of C/EBPs in G-MDSCs and M-MDSCs and among various differentiation stages.

The C/EBP family may differentially regulate MDSCs from neutrophils and monocytes. Although all members of the C/EBP family are required for the differentiation of neutrophils and monocytes, they are modestly expressed. During pathological conditions, C/EBPα may be downregulated in MDSCs, but C/EBPβ, C/EBP-δ, CHOP may be strongly upregulated.

Most of the findings discussed above are from tumor-derived MDSCs or from MDSCs in an inflammation setting. Inflammation and hypoxia are two conditions closely associated with tumor development (50, 51), and the C/EBP family may have similar mechanisms regulating MDSCs in tumor and inflammation settings. We hope to use existing evidence to provide a predictive description of the C/EBP family in MDSCs during pathological conditions, especially in the tumor microenvironment.

The molecular mechanisms of the C/EBP family in MDSCs have not been fully elucidated, especially with regard to cooperation among family members or with other TFs, cytokines, proteins, and microRNAs. Although the precise mechanisms require further study, the broad effects of MDSCs render them an ideal therapeutic target, especially for immune checkpoint inhibitor (ICI) treatment. For example, ipilimumab treatment decreases levels of M-MDSCs and elevates those of CD8 effector memory T cells in advanced melanoma (58). Ibrutinib reduces the generation and function of MDSCs, significantly enhancing the efficacy of anti-PD-L1 therapy in a murine breast cancer model (59). Additionally, entinostat (ENT) efficiently inhibits MDSC function in combination with ICI treatment (60). The proposed mechanism involves inhibition of STAT3 activation, and such inhibition by the tyrosine kinase inhibitor sunitinib in tumor-bearing mice also reduces MDSC numbers (61). Moreover, omaveloxolone (RTA 408) suppresses MDSC functions, inhibiting the NF-κB kinase subunit at high concentrations (62, 63). Some studies have considered STAT3 to be an ideal target. Other TFs, such as C/EBPβ and NF-κB, might also become prospective therapy targets, as previously achieved via conditional genetic ablation of the Cebpb gene. Overall, much deeper insight is urgently needed.

## Author Contributions

WW drafted the manuscript and designed the figures. XX reviewed the manuscript structure and ideas. LM evaluated and reviewed manuscript structure, ideas, and science. SW conceived the topic and revised the manuscript. All authors read and approved the final manuscript.

### Conflict of Interest Statement

The authors declare that the research was conducted in the absence of any commercial or financial relationships that could be construed as a potential conflict of interest.
